# Ubiquitous distribution of salts and proteins in spider glue enhances spider silk adhesion

**DOI:** 10.1038/srep09030

**Published:** 2015-03-12

**Authors:** Gaurav Amarpuri, Vishal Chaurasia, Dharamdeep Jain, Todd A. Blackledge, Ali Dhinojwala

**Affiliations:** 1 Department of Polymer Science, The University of Akron, Akron, OH 44325; 2 Department of Mechanical Engineering, The University of Akron, Akron, OH 44325, USA; 3 Department of Biology, Integrated Bioscience Program, The University of Akron, Akron, OH 44325, USA

## Abstract

Modern orb-weaving spiders use micron-sized glue droplets on their viscid silk to retain prey in webs. A combination of low molecular weight salts and proteins makes the glue viscoelastic and humidity responsive in a way not easily achieved by synthetic adhesives. Optically, the glue droplet shows a heterogeneous structure, but the spatial arrangement of its chemical components is poorly understood. Here, we use optical and confocal Raman microscopy to show that salts and proteins are present ubiquitously throughout the droplet. The distribution of adhesive proteins in the peripheral region explains the superior prey capture performance of orb webs as it enables the entire surface area of the glue droplet to act as a site for prey capture. The presence of salts throughout the droplet explains the recent Solid-State NMR results that show salts directly facilitate protein mobility. Understanding the function of individual glue components and the role of the droplet's macro-structure can help in designing better synthetic adhesives for humid environments.

During prey capture by an orb spider, a flying insect impacts the web and is retained by the sticky strands of capture silk that are composed of flagelliform-fibers coated with uniformly spaced glue droplets^1^. These adhesive glue droplets therefore play a critical role in spider's hunting success by giving the spider time to subdue the prey^2^,^3^,^4^. The glue material acts as a viscoelastic solid over short time-scales so that its adhesive force is proportional to the rate of pull, allowing the glue to adhere strongly to the fast moving prey. The glue also acts as an elastic material over long time-scales, such that the glue can support subdued prey for a long period of time^5^.

Spider glue adhesion is humidity responsive^6^,^7^,^8^,^9^. While most synthetic adhesive systems are negatively affected by increased humidity^10^, viscid-silk glue generates higher adhesion forces as humidity increases, and in some species adhesion continues to improve at conditions close to 100% relative humidity (R.H.)^8^. Therefore, understanding the glue composition and its adhesion mechanism can provide a framework to design synthetic adhesives that function in humid environments.

The composition of the glue droplet includes glycoproteins, water, and low molecular weight organic salts and polar aliphatic compounds, collectively referred to here as salts^4^,^11^,^12^,^13^. The glycoproteins are composed of two O-glycosylated proteins, ASG-1 and ASG-2^14^. N-acetylgalactosamine sugar has also been detected in the glycoproteins^4^. Salts found in the glue droplet are hygroscopic and are hypothesized to absorb and retain atmospheric water in the glue droplet^4^,^11^,^13^. Recently, Solid-State NMR investigations showed that the salts directly facilitate adhesion by solvating the glycoproteins^15^.

Optically, an immobilized glue droplet shows a heterogeneous structure with a distinct granular core region surrounded by a transparent sheet-like region^16^. Two models have been outlined in the literature to explain the structural heterogeneity. The first model suggested a concentrated glycoprotein core surrounded by a shell region containing salts and water^4^. The glycoproteins present in the core region were hypothesized to be primarily responsible for adhesion. However, Opell et al.^17^ found little correlation between the size of the visible glycoprotein core and silk adhesion. They suggested a new model wherein the glycoprotein core acts as an anchor to the fiber, while an additional layer of optically transparent glycoprotein glue, present between the glycoprotein core and the shell region, is responsible for adhesion^8^,^17^. The glycoprotein glue transfers load onto the axial fiber by elongating and resisting slippage. However, both models are based on optical and physical measurements, and lack chemical evidence of heterogeneity in structure. Moreover, the recent finding that salts facilitate protein mobility at a molecular level^15^ suggests an alternative model where both salts and proteins are distributed ubiquitously throughout the glue droplet.

To test these competing hypotheses, we use confocal Raman microscopy to construct a three-dimensional chemical map of salts and proteins in a glue droplet. Raman spectroscopy has been used on natural silk fibers to characterize protein structure and conformation^18^,^19^,^20^,^21^,^22^,^23^, however it has never been used for the chemical mapping of a spider glue droplet.

Before prey capture, the viscid silk glue droplet, referred to here as pristine-silk, is in the form of a suspended droplet on the fiber (Figure 1A), and after prey capture, the glue droplet is spread thinly on the surface (Figure 1C). To replicate this prey capture process, pristine-silk was probed in two forms: suspended, and immobilized on a substrate. Additionally, the pristine-silk was washed with deionized water to separate the salts from the proteins^11^,^15^. The resulting solution containing the salts is hereafter referred to as wash-residue, and the remaining glue proteins are referred to as washed-glue.

Nature commonly uses variation in spatial arrangement of chemical components to create multiple functionalities. For example, marine mussels employ spatial variation in proteins and cross-linking density to generate strong under-water adhesion^24^,^25^. Given the visually heterogeneous structure of the spider glue droplet (Figure 1C), spatial mapping of the chemical components in the glue droplet, before and after immobilization, can help test the divergent predictions of the silk macro-structure and its role in silk adhesion.

## Results and Discussion

### Optical Microscopy

**Figure 1:**
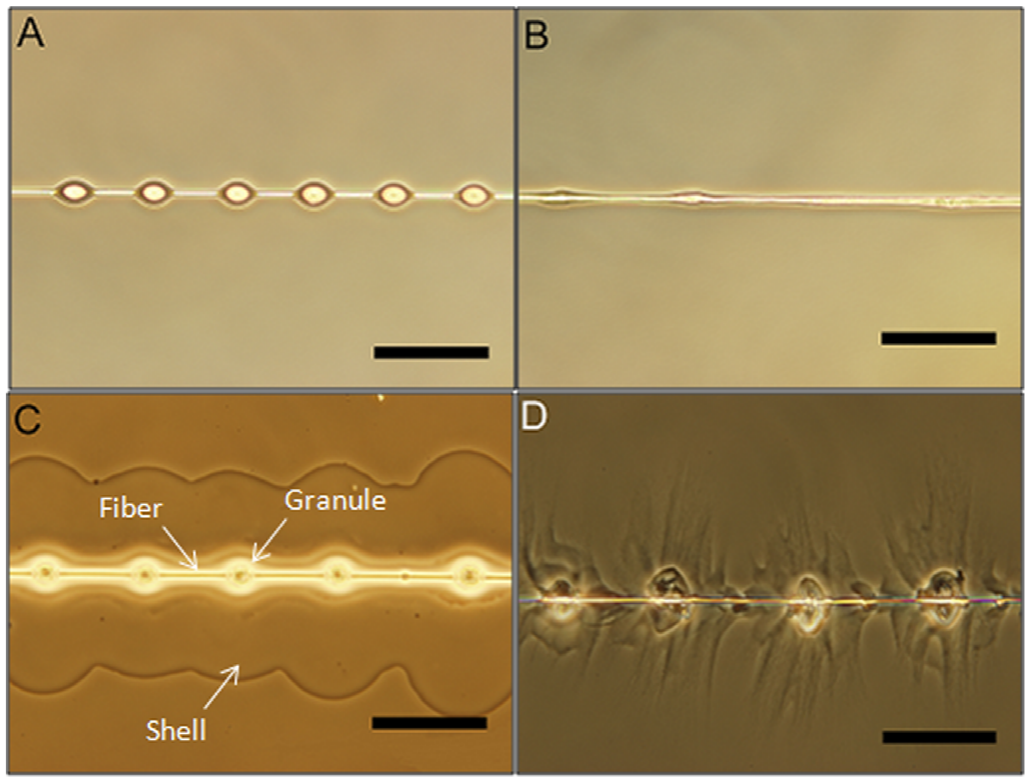
Effect of washing pristine silk with de-ionized water in suspended (A and B) and immobilized state (C and D). The silk was immobilized on cleaned glass substrate (see methods). All scale bars are 100 *µ*m.

Pristine-silk in suspended form shows optically homogeneous glue droplets (Figure 1A). The bright oval region in the center is due to reflection of transmitted light on a convex shaped droplet, as shifting the focal plane changes the size of the bright oval region. The pristine droplet spreads to form optically distinct regions upon immobilization on a glass substrate (Figure 1C). The flagelliform fiber is surrounded by a circular granular region, referred to here as the granule, followed by a more fluid-like region, referred to here as the shell. The shell regions from adjacent glue droplets coalesce to form a continuous sheet that spreads all around the fiber.

Similar regions are observed upon washing the immobilized pristine silk with de-ionized water (Figure 1D). The granule region appears enlarged and spread, while the shell region shows a distinct wrinkling pattern. This wrinkling pattern is a signature of solid and elastic-like material left behind after washing. Washing the glue droplet removes all the aqueous salt content, leaving behind only the glycoproteins^11^,^15^. Observing the wrinkling pattern in the shell region after washing, we therefore conclude that glycoproteins are present in the shell region.

Washing suspended pristine droplets produced an irregular pattern (Figure 1B). In some places, the granular region appeared collapsed on the fiber, while in other places the uniform circular cross-section of the fiber was observed. We suspect that the surface tension of water during drying spreads the granule over the fiber^26^.

### Raman spectroscopy

Pristine-silk contains both salts and proteins, but after washing, the washed-glue contains only proteins, while the wash-residue contains only salts^11^,^15^. The Raman peaks specific to salts and proteins can be identified by comparing the Raman spectra of these three samples: pristine-silk, washed-glue, and wash-residue. Please note that the Raman spectra of these three samples predominantly includes signal from the glue components and not from the flagelliform fiber. The Raman spectra of the flagelliform fiber is included in Figure S7.

**Figure 2:**
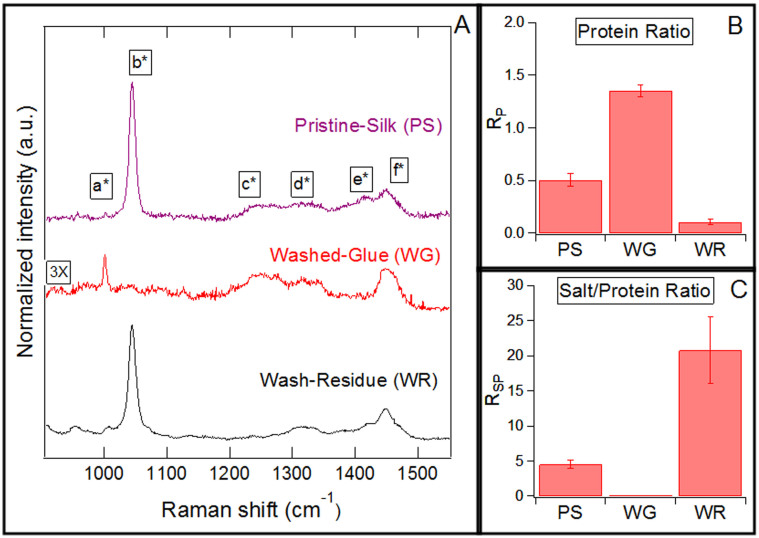
(A) Raman spectra of pristine-silk, washed-glue and wash-residue. Pristine-silk was in suspended state, while the washed-glue and wash-residue were immobilized on a CaF_2_ substrate. Labeled Raman peaks and their assignments are: a* - Phenylalanine^32^, b* - Isethionic Acid^21^, c* - Amide-III^23^, d* - Alanine^32^, e* - Glycine^21^, f* - CH_3_ asymmetric bend/CH_2_ bending^32^. (B) R*_P_* quantifies the spectral differences and is defined as the ratio of area of protein-specific peaks c* to the area of common peak d*. (C) R*_SP_* is the ratio of area of salt-specific peak b* to the area of protein-specific peaks c*.

In Figure 2A, the sharp peak labeled b* is absent in washed-glue, but is present in pristine-silk and wash-residue. In the literature, the peak b* is assigned to SO_3_ stretching mode^21^. The SO_3_ group is present in three salts (isethionic acid, n-acetyl taurine, and taurine) that constitute a significant proportion of salts present in the glue of *Larinioides cornutus*^15^. The absence of the peak b* in washed-glue, and its presence in pristine-silk and wash-residue, demonstrates that this peak is a salt-specific peak.

On the other-hand, the peak c* is present in pristine-silk and washed-glue but negligible in wash-residue. The peak c* is assigned to two amide-III bands of proteins^21^,^23^. To quantify the spectral differences, we define the ratio R*_P_* as the area of peak c* to peak d* (SI1 text). Peak d* is assigned to Alanine^21^, an amino acid that is present in both the salts and the proteins. Hence, R*_P_* signifies the proportion of proteins in the sample. In Figure 2C, as expected, the value of the ratio R*_P_* is almost zero in the wash-residue, which contains only salts, and the ratio is highest in the washed-glue, which contains only proteins (Figure 2C). The ratio R*_P_* is thus used as a metric to determine the presence of protein.

The spectroscopy data can also be used to calculate the ratio of salts to protein in the sample. Ratio R_SP_ is defined as the area of salt-specific peak b* to protein-specific peak c* (SI1 text). This ratio determines the spatial variation in the relative content of salts vs. proteins in different regions of the glue droplet. NMR studies show that the three salts corresponding to peak b* are present in significant amounts in the glue of various spider species^13^. Hence, we treat peak b* as representating salts. As expected, R_SP_ is close to zero for washed silk since there are no salts left in washed-glue^15^.

### Pristine-silk glue droplet in suspended state

**Figure 3:**
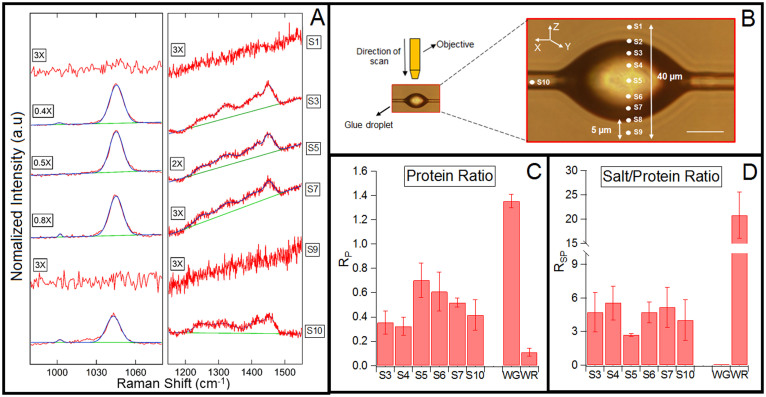
Chemical composition at different depths of a suspended pristine-silk glue droplet using z-scan confocal Raman spectroscopy. (A) Raman spectra with the fitting function for regions S1, S3, S5, S7, S9, and S10. The spectral intensity was normalized by multiplying with a constant denoted on the left of each spectra. (B) Experiment set-up: A pristine-silk glue droplet suspended under a 100X objective. The focal plane was changed in steps of 5 *µ*m to probe regions labeled S1-S9. S3 and S7 are in the peripheral region. S4–6 are the core region. S10 is on the thread outside the glue droplet. Scale bar is 100 *µ*m. (C) R*_P_* quantifies the spectral differences and is defined as the ratio of the area of protein-specific peaks c* to the area of common peak d*. (D)R*_SP_* is the ratio of the area of salt-specific peak b* to the area of protein-specific peaks c*. n = 5.

Confocal Raman microscopy was used to do a z-scan of the suspended pristine-silk glue droplet (Figure 3B). The spatial (xy) and axial (z) resolution of the laser spot size was less than 3 *µ*m (SI2 text). The droplet's axis was aligned in the center of the XY plane and the edge of the droplet was kept in focus to probe the core region, denoted as S5. The z-axis was varied in steps of 5 *µ*m to probe regions above and below the core region. Depending on the initial position of S5, the location of the regions probed may vary among different droplets or due to refraction of laser light. However, the relative position of probed regions as shown in Figure 3B was kept consistent in all the z-scan experiments. The flagelliform fiber was also probed and denoted as S10.

Figure 3A shows the Raman spectra and the fitting function for the peripheral regions (S1, S3, S7, S9), core region (S5), and thread region (S10) of the droplet. Since the intensity of peak b* was significantly greater than the intensity of peaks c* and d*, the spectra was deconvoluted in two parts (SI1 text). Regions S1 and S9 are ~ 5 *µ*m outside the glue droplet, and hence as expected, no signal was detected in these regions. The slope in the Raman spectra is due to fluorescence of the glue components. Also, the Raman signal in the peripheral regions S2 and S8 were not analyzed due to low signal to noise ratio.

Early glue-structure models^4^,^17^, based mainly on optical microscopy, predicted the salts to be present primarily in the peripheral regions. However, the salt-specific peak b* was detected throughout the droplet, including the core region, S4–S6. Furthermore, the protein signal was detected throughout the droplet, including the peripheral region, S3 and S7. Ratio R*_P_* was between 0.3–0.7 (Figure 3C), confirming the presence of protein throughout the glue droplet. The R*_P_* values in region S3–S7 were significantly higher than the R*_P_* value of wash-residue (salts only). Thus, both salts and proteins are clearly distributed throughout the glue droplet and in relatively similar proportions. However, the current data-set cannot resolve if the absolute amount of salts or proteins varies spatially in the droplet.

Figure 3D shows the values of R*_SP_* as we scan across the glue droplet. We expect this ratio to be very high for wash-residue and almost zero for washed-glue, because the salts are highly water soluble. The R*_SP_* values observed in the core and the peripheral regions of pristine-silk glue droplet are significantly different than the values observed in the washed-glue and wash-residue. These results imply an ubiquitous presence of salts and proteins throughout the glue droplet. The values of R*_S_* and R*_SP_* as a function of the depth in the droplet are not statistically different, but the large variance means that we cannot determine if the distribution of salts and proteins is homogeneous across the droplet.

Presence of salts in the core region explain the humidity dependent volume changes observed in the granular region of the glue droplets^6^,^7^,^8^. The presence of hygroscopic salts in the core makes the core region increase in volume with an increase in humidity. Proteins are clearly present in the flagelliform fiber, S10, however the presence of salts is probably due to the thin coating of glue left behind after the formation of glue droplets due to Rayleigh instability^4^,^27^.

### Pristine-silk glue droplet in immobilized state

**Figure 4:**
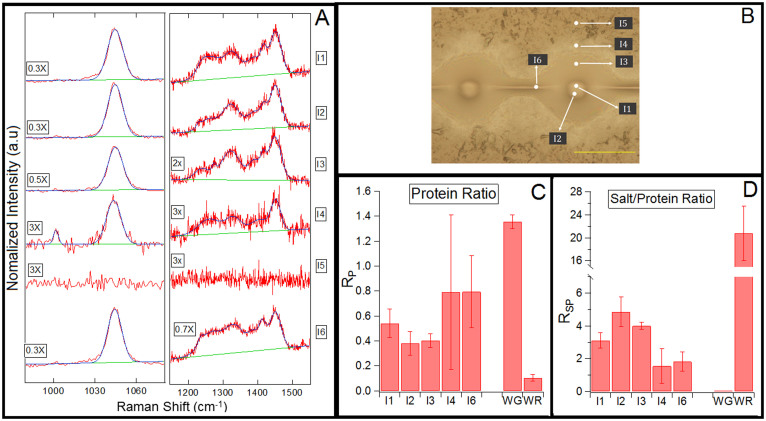
Chemical composition at different regions across an immobilized pristine-silk glue droplet using confocal Raman spectroscopy. (A) Raman spectra with the fitting function of different regions, I1-I6, of immobilized pristine silk. The spectra intensity is normalized by multiplying by a constant denoted on the left of each spectra. (B) Pristine-silk glue droplet immobilized on a CaF_2_ substrate. Raman spectra collected at regions marked as I1-I6. Scale bar is 100 *µ*m. (C) R*_P_* quantifies the spectral differences and is defined as the ratio of the area of protein-specific peaks c* to the area of common peak d*. (D) R*_SP_* is the ratio of the area of salt-specific peak b* to the area of protein-specific peaks c*. n = 6.

Our observation of the ubiquitous distribution of salts and proteins in the suspended pristine-silk glue droplet is surprising given that a discrete granule is clearly visible at the center of immobilized droplets. Therefore, we immobilized a glue droplet on a CaF_2_ substrate to probe its visually heterogeneous structure at six different locations (Figure 4B). The Raman spectra for wavenumber regions specific to salts and proteins are shown in Figure 4A. The spectra in region I5 was collected away from the glue droplet to confirm that none of these Raman peaks were due to the CaF_2_ substrate. The ratios, R*_P_* (Figure 4C) and R*_SP_* (Figure 4D), are very similar to those observed for pristine-silk in suspended state. Similar to the pristine-silk in suspended state, R*_P_* and R*_SP_* values across the immobilized droplet are significantly different than the respective ratio values of washed-glue and wash-residue. The presence of proteins in the peripheral regions I3 and I4 is consistent with the observation of wrinkles in this region after washing away the salts (Figure 4D). The results demonstrate that both salts and proteins are present across the immobilized glue droplet.

Based on visual observations of a darker core region in the immobilized glue droplet (Figure 1C), previous glue structure models^4^,^17^ concluded that the salts and proteins are likely segregated in the glue droplet. Our Raman data conclusively demonstrates that the salts and proteins are present throughout the glue droplet. However, the observation of a darker core is still puzzling, so we next test the hypothesis that the visible core results from spatial variation in protein composition.

### Washed Glue

Spider glue contains two proteins: ASG-1 and ASG-2. The amino acid sequence of ASG-1 includes regions similar to chitin-binding proteins, and that of ASG-2 includes regions similar to flagelliform fiber^14^. Functionally, ASG-1 acts as an adhesive, while ASG-2 acts as a cohesive. Hence, we expect ASG-1 protein to reside in the shell region to bind with the substrate, while ASG-2 protein to reside in the core region, to provide an elastic anchorage on the flagelliform fiber. Alternatively, both proteins may be broadly intermingled to provide a balance between adhesion and cohesion throughout the droplet. cDNA data predicts significant differences in the proportion of the amino-acid glycine in the two glue proteins (3.7% in ASG-1 and 16.4% in ASG-2)^14^ compared to the flagelliform protein (54.7%)^28^ (SI3 text). Although, there were other differences in the amino-acid composition, we focus on glycine for comparison because it is Raman active and has a distinct peak in the Raman spectrum. Hence, a spatial Raman scan of immobilized washed-glue, containing only proteins and no salts, was conducted to test the hypothesis that spatial variation in ASG-1 and ASG-2 distribution produces visual heterogeneity in the immobilized glue droplet.

**Figure 5:**
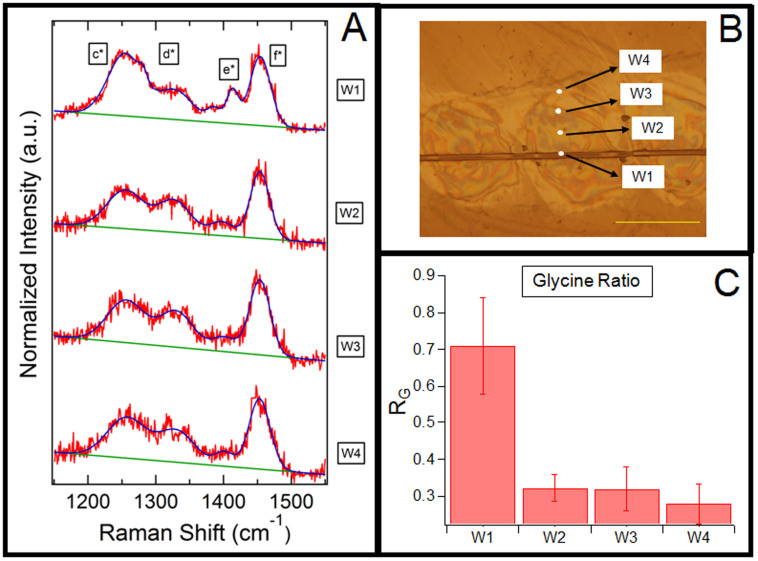
Chemical composition at different regions of the washed-glue droplet using confocal Raman spectroscopy. (A) Raman spectra with the fitting function of different regions, W1-W4, of immobilized washed-glue. (B) Immobilized washed-glue droplet on CaF_2_ substrate. Raman spectra collected at the regions marked as W1-W4. Scale bar is 100 *µ*m. (C) R*_G_* quantifies the spectral differences and is defined as the ratio of the area of glycine-specific peak e* to the area of common peak d*. n = 4.

Figure 5B shows four different locations probed on the washed-glue sample immobilized on a CaF_2_ substrate (see methods). Region W1 corresponds to the flagelliform fiber at the center of the glue droplet, and the regions W2-W4 are located radially at 5 *µ*m increments from W1. The differences in the Raman spectra are shown in Figure 5A. Peak e*, assigned to Glycine^21^, is significantly pronounced in W1, flagelliform fiber, which is abundant in Glycine content^28^. However, the Raman spectra in region W2-W4 are nearly identical, despite clear differences in the amino acid composition of the ASG-1 and ASG-2 glue proteins. To quantify the differences, we defined ratio R*_G_* as the ratio of the area of Glycine peak (e*) to the area of the Alanine peak (d*). Figure 5C shows that the ratio R*_G_* is significantly different in the fiber region (W1) but similar across the glue regions (W2-W4). Raman spectra at lower wavenumbers are provided in supplementary information (SI5). This result suggests that ASG-1 and ASG-2 are broadly mixed within the glue droplet, at least at levels resolvable by Raman spectroscopy. ASG-1 is predicted to be responsible for adhesion, while ASG-2 is responsible for elasticity. The total work of adhesion is a product of surface adhesion and bulk cohesion^29^. Therefore, the greatest adhesion occurs when both surface and bulk forces are optimized. Hence, ubiquitous distribution of the glue proteins, ASG-1 and ASG-2 across the glue droplet makes spider glue a functional adhesive.

### Ubiquitous presence of salts and proteins

Our data supports a revised structural model for glue-droplets where salts and proteins are present ubiquitously throughout the glue droplet. The presence of salts and proteins across the glue droplet provides several advantages to glue function. First, adhesion forces act over the entire volume of the glue droplet, not just in the core region. Thus, insects need not penetrate deeply into the shell region to come in contact with the core region for the ASG-1 or ASG-2 proteins. The entire surface area of the glue droplet is an adhesive, and hence, even a tangential graze by an insect on the sticky surface of the glue droplet can result in prey capture. This is physically observable by gently grazing a fine-tipped probe on a suspended glue droplet. The gentle tugging at the surface resulted in drawing of fibrous threads from the droplet (shown in Figure S8).

Second, an ubiquitous presence of salts and proteins, in similar ratios in both suspended and immobilized pristine-silk, supports a critical role of salts in solvating glycoproteins. Capture thread adhesion is significantly reduced after water washes the salts away^15^. Solid-State NMR shows that on a molecular level, removal of salts results in an irreversible collapse of the protein structure^15^. Thus, salts not only sequester atmospheric water but also maintain and solvate the protein molecules. Yet, this solvation only happens if salts are broadly distributied throughout the droplet.

Third, on a molecular scale, both ASG-1 and ASG-2 glue proteins are likely present throughout the droplet. cDNA data predicts domains in ASG-1 that are similar to adhesive proteins, while ASG-2 shows domains similar to cohesive proteins^14^. The presence of these functionally diverse proteins throughout the droplet suggests a balance of cohesive and adhesive forces that makes viscid glue a superior adhesive^29^. Future studies should investigate if there is fine scale segregation of these two proteins that might result in a cohesive gradient as seen in other biological adhesives, such as marine-mussels^24^.

Finally, our confocal Raman data clearly shows that the visual heterogeneity in the glue droplet (Figure 1C) is not due to simple segregation of salts and proteins. Even the two glue proteins, ASG-1 and ASG-2 are likely present throughout the droplet. Thus, the question remains, what causes the visual heterogeneity? We hypothesize that this difference could be due to the variation in water content in the core and peripheral regions of the glue droplet. One of the reasons for the differntial water content could be variation in crosslinking density which leads to variation in swelling. Understanding the process by which this crosslinking is achieved could provide key insights into the design of synthetic adhesives for humid environments.

## Methods

### Web collection

Naturally spun orb-webs of *Larinioides cornutus* were collected in Akron, OH, USA at night. To procure webs, a square cardboard frame with an inner dimensions 10 cm × 10 cm was softly pressed against the suspended web and a soldering iron was used to cut the silk threads around the frame and thus obtain an undisturbed section of the web. No additional adhesives were used to hold the thread to the cardboard. In order to remain consistent and limit within web variation, only regions directly below the spider's hub were collected. The procured orb webs were stored in laboratory environment at 22 ± 2°C and 30 ± 5% relative humidity (RH) and tested over a period of six months. No significant difference was observed in the Raman spectra of aged and fresh samples.

### Sample preparation

The viscid silk threads were immobilized on glass substrate for optical microscopy, and on CaF_2_ substrate for Raman spectroscopy. Threads were transferred from the web to the substrate using a two pronged fork. The substrates were cleaned by the following protocol: 10 min in a base bath (pH~14) followed by washing with copious amount of de-ionized (DI) water, sonication in acetone, chloroform, methanol and DI water for 15 min each, drying in N_2_ environment, followed by 10 min of oxygen pulse-plasma treatment. The silk samples were conditioned for two days in 60% RH before immobilizing on the substrate. The glue droplet has been reported to remain sticky over a period of one year^30^,^31^. No significant difference was observed in the spreading behavior of aged and fresh silk glue droplets upon immobilization.

### Washing

The silk threads were immersed in a beaker of DI water and held stationary for 5 minutes. After washing, the samples were immediately moved to a desiccator with P_2_O_5_ pellets and dried overnight. The washed away components were retrieved by concentrating the washed solution^15^.

### Optical Microscopy

Olympus BX51 microscope with phase contrast lenses (Olympus UPlanFL) was used for capturing optical images. Samples were imaged before and after washing.

### Raman spectroscopy

Raman spectra were recorded at 22 ± 0.5°C at a 30 ± 5% RH using a LabRam HR Micro Raman Spectrometer (Horiba) coupled to a Olympus BX41 motorized stage microscope. The 532 nm line of Nd:YAG laser beam was focused using a 100X objective (0.9 NA, Olympus), generating an intensity of 1.5 mW at the sample. The entrance slit of the monochromator was fixed at 100 *µ*m. The confocal aperture of the monochromator was kept at 50 *µ*m. In confocal microscopy, aperture is used to improve depth resolution. However, this comes with a trade-off by reducing the magnitude of the Raman signal. In our study, the spatial resolution was more important for mapping the Raman spectra across the glue droplet. Hence, we chose a low confocal aperture of 50 *µ*m to get a depth resolution of 2 *µ*m (SI2 text).

Data were collected using a peltier-cooled CCD detector (1024 × 256 pixels). Samples were irradiated for 5–15 min to stabilize the signal and quench fluorescence before spectra acquisition. Measurement time for a single spectrum varied between ~15–40 min to obtain the best signal-to-noise ratio. We tested for laser damage due to prolonged exposure, and no sign of sample degradation was observed (SI4 text). The number of glue droplets probed for suspended pristine-silk, immobilized pristine-silk, and washed-immobilized silk was 5, 6, and 4, respectively. The glue droplets were collected from the webs of different adult-female spider individuals. Data were analyzed using IGOR's multi-peak fitting function. Selected regions were fitted using a linear baseline and gaussian line-functions (SI1 text).

## Author Contributions

G.A. and V.C. performed the experiments and collected data. G.A., V.C., D.J., T.A.B. and A.D. analyzed the data. G.A., V.C., D.J., T.A.B. and A.D. wrote the manuscript. All authors discussed the results and commented on the manuscript.

## Supplementary Material

Supplementary Information Supplementary Information
