# Supplementary material for: Ubiquitous distribution of salts and proteins in spider glue enhances spider silk adhesion
Source: Sci Rep. 2015 Mar 12;5:9030. doi: 10.1038/srep09030 (PMC4357010; doi:10.1038/srep09030)
Supplement: Supplementary Information [file srep09030-s1.pdf]

Ubiquitous distribution of salts and proteins in spider glue enhances spider silk adhesion

Gaurav Amarpuri,[†] Vishal Chaurasia,[‡] Dharamdeep Jain,[†] Todd A. Blackledge,[¶] and Ali Dhinojwala[*,†]

Department of Polymer Science, The University of Akron, Akron, OH 44325, USA, Department of Mechanical Engineering, The University of Akron, Akron, OH 44325, USA, and Department of Biology, Integrated Bioscience Program, The University of Akron, Akron, OH 44325, USA

E-mail: ali4@uakron.edu

[*]To whom correspondence should be addressed
[†]Department of Polymer Science, The University of Akron, Akron, OH 44325, USA
[‡]Department of Mechanical Engineering, The University of Akron, Akron, OH 44325, USA
[¶]Department of Biology, Integrated Bioscience Program, The University of Akron, Akron, OH 44325, USA

SI1: Peak-fitting

The Raman spectra was deconvoluted using IGOR's multi-peak fitting function. The following peak positions were used during fit optimization:

Table S1: Initial input wavenumbers used for peak-fitting for pristine-silk, washed-glue and wash-residue samples

S.No.	Region	Assignement	Raman Wavenumber (cm^{-1})
1	a*	Symmetric SO_3 stretch	1045
2	b*	Amide-III	1240, 1265
3	c*	Alanine	1330
4	d*	Glycine	1416
5	e*	CH_3 asymmetric bend / CH_2 bending	1452

The position, width and height of peaks were allowed to vary during fitting optimization. In the final fit function obtained, the wavenumbers varied ~2-15 cm^{-1} from the input wavenumber. The position and width of the peak at region c* was fixed for the purpose of quantification of the spectra profile, only in case of washed-silk, where the presence of peak c* was minimal.

The slope in the baseline of the spectra is due to fluorescence of the glue components. However, no significant difference in the values of ratio R_P and R_{SP} was observed due to fluorescence.

SI2: Laser spot resolution

The axial resolution of the laser spot was experimentally determined by conducting a z-scan on a ∼1μm thick polystyrene (PS) film coated on glass substrate. Figure S1 shows change in Raman signal in the hydrocarbon region at different depths of probe. L0 is the Raman spectra of the surface of the PS film. The focal plane was moved in steps of 1μm above (L1 and L2) and below (L-1, L-2 and L-3) the surface of PS film. L-1 is expected to be at the interface of PS and glass substrate.

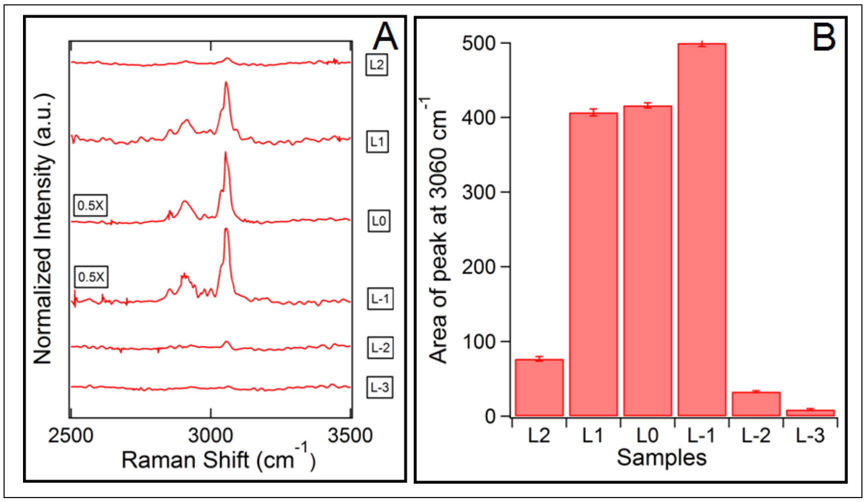

Figure S1: Laser spot resolution. A) Raman spectra at different depths of PS film coated on a glass substrate. B) Area of Raman peak at 3060 cm^{-1} for different depths. Significant drop in intensity is observed at different probe depth.

Significant drop in signal is detected at 2μm above the film surface, L2, and 1μm below the expected film interface with CaF_2, L-2. Hence, the laser spot size resolution was measured as ∼3μm.

The spatial resolution (x and y) of the laser spot is determined by formula (1).

$$D = \frac{1.22 * \lambda}{NA} \tag{1}$$

For wavelength (λ) 532 nm and 100X objective with a numerical objective (NA) of 0.9, the spatial laser spot was 0.72 μm

SI3: Amino-acid composition

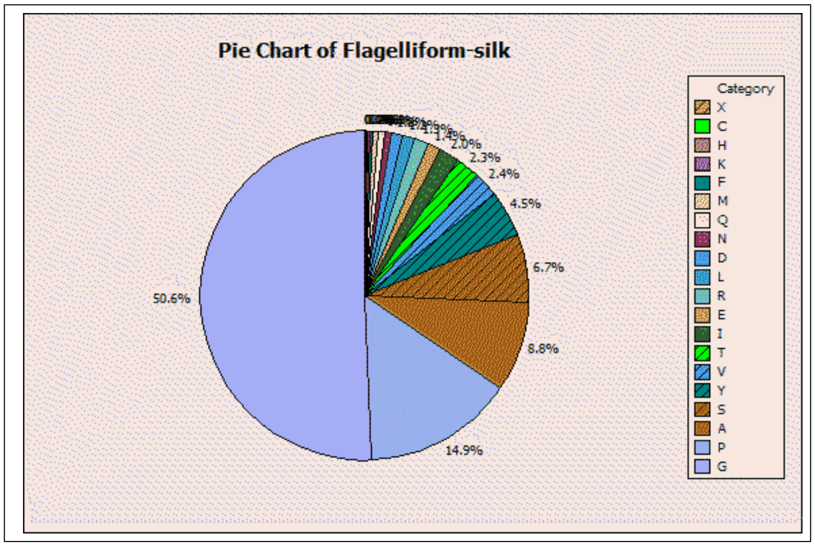

Figure S2: Composition of amino-acids in the flagelliform fiber protein from *Nephila clavipes*. The amino acid sequence was obtained from the GeneBank under accession number AH009147.1.

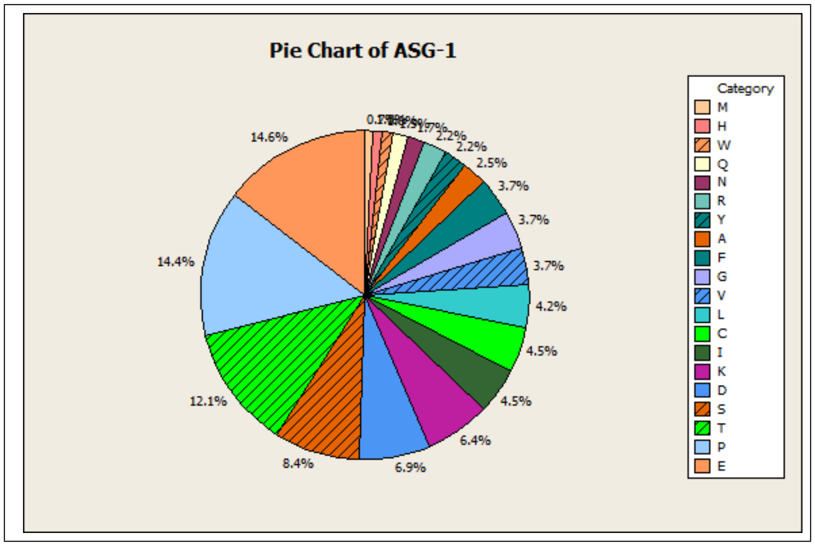

Figure S3: Composition of amino-acids in the ASG-1 glue proteinfrom *Nephila clavipes*. The amino acid sequence was obtained from the GeneBank under accession number EU780014.

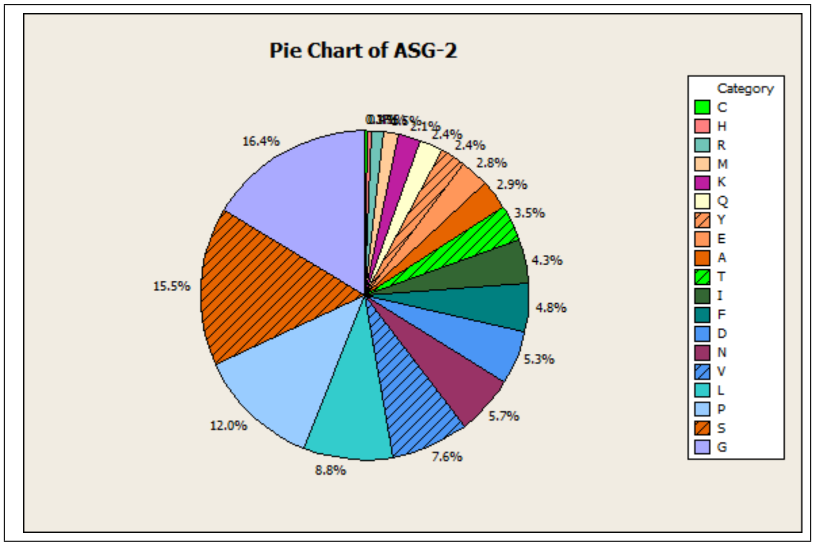

Figure S4: Composition of amino-acids in the ASG-2 glue protein from *Nephila clavipes*. The amino acid sequence was obtained from the GeneBank under accession number EU780015.

SI4: Effect of prolonged laser exposure

To test the effect of prolonged laser exposure, we recorded the Raman spectra of the same region of washed-glue under short and long exposure time. We followed the experimental conditions (laser power and exposure duration) listed in the Lefvre, 2012 article, to obtain the Raman spectra over short-duration. This spectrum was then compared to the spectrum obtained using the experimental conditions listed in our manuscript (Figure S5). No significant effect in the Raman spectra profile was observed upon increasing the exposure time. Also, no physical damage was observed.

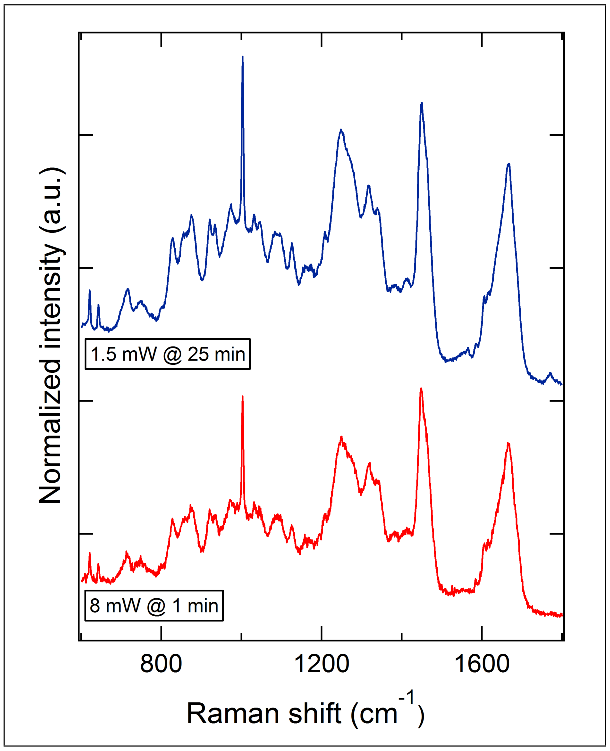

Figure S5: Effect of exposure time on the Raman spectra of the washed-silk.

SI5: Extended range Raman spectrum of Washed-Glue

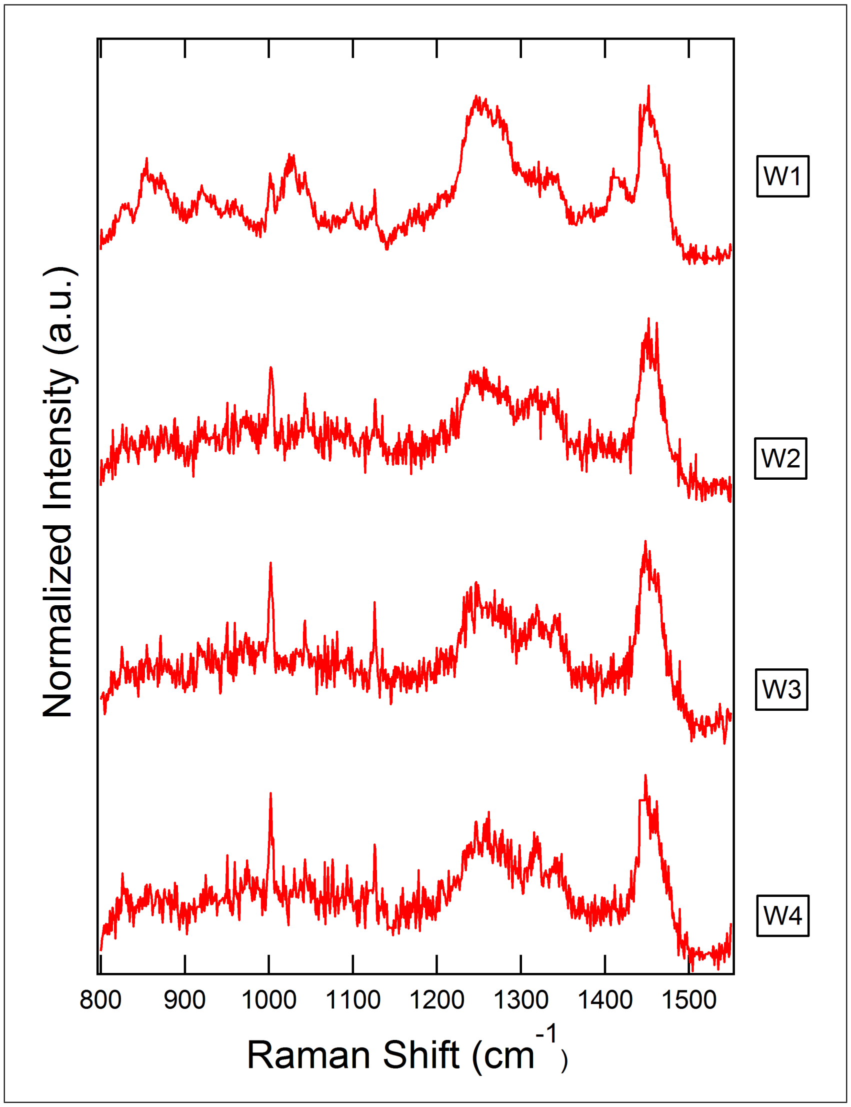

Figure S6: Extended range Raman spectrum of washed-glue: As described in the main article, Region W1 corresponds to flagelliform fiber and W2-W4 corresponds to different regions of the washed-glue (Figure 5 in the main article). Notice the difference in the Raman spectra of W1 and W2-4 in the region 800-1100 cm^{-1}. The difference in the Raman peaks can be attributed to the differences in the amino-acid composition of the flagelliform fiber and the glue.

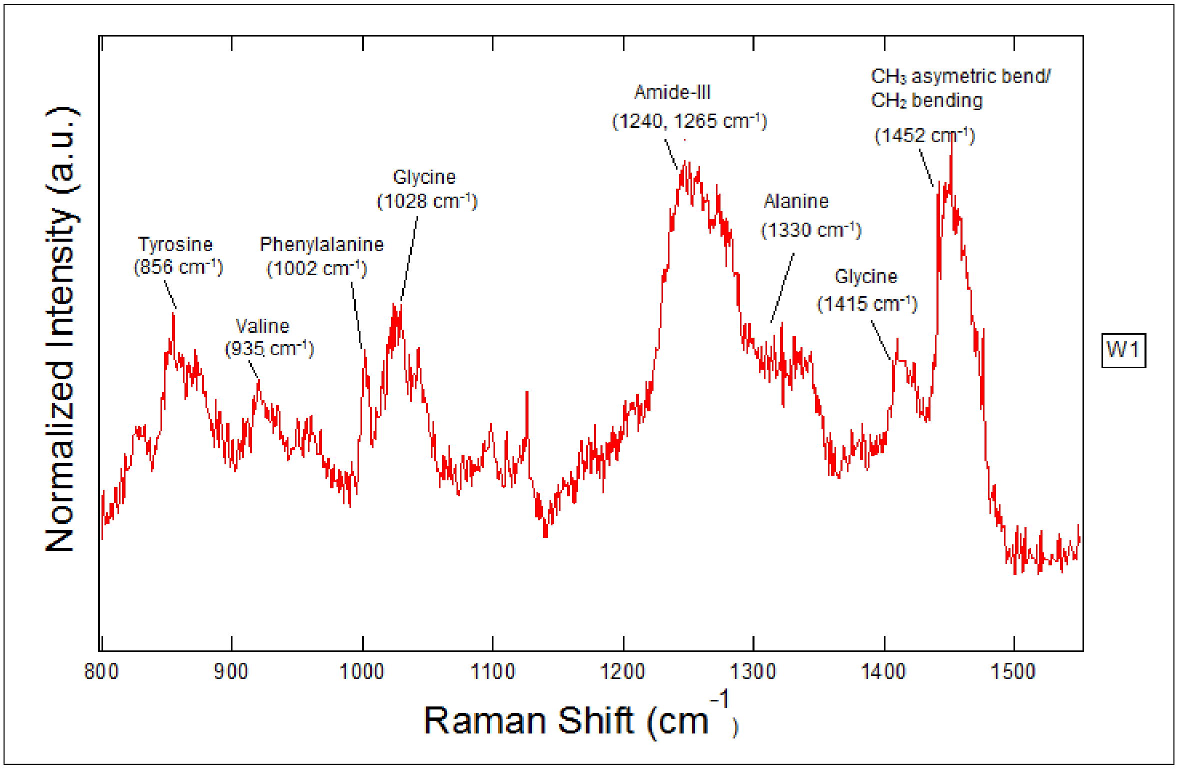

Figure S7: Raman spectrum of flagelliform fiber (Region W1 in Figure 5 of the main article). The Raman peaks have been assigned in the corresponding amino acids and chemical bonds.

SI6: Probing the surface of an orb-web glue droplet

The surface of an orb-web glue droplet was probed using a fine-tipped probe. Interestingly, the glue droplet was sticky even when the tip just grazed the surface of the glue droplet. Notice the puling of fibrous threads from the surface of the glue droplet in the Figure S8-D.

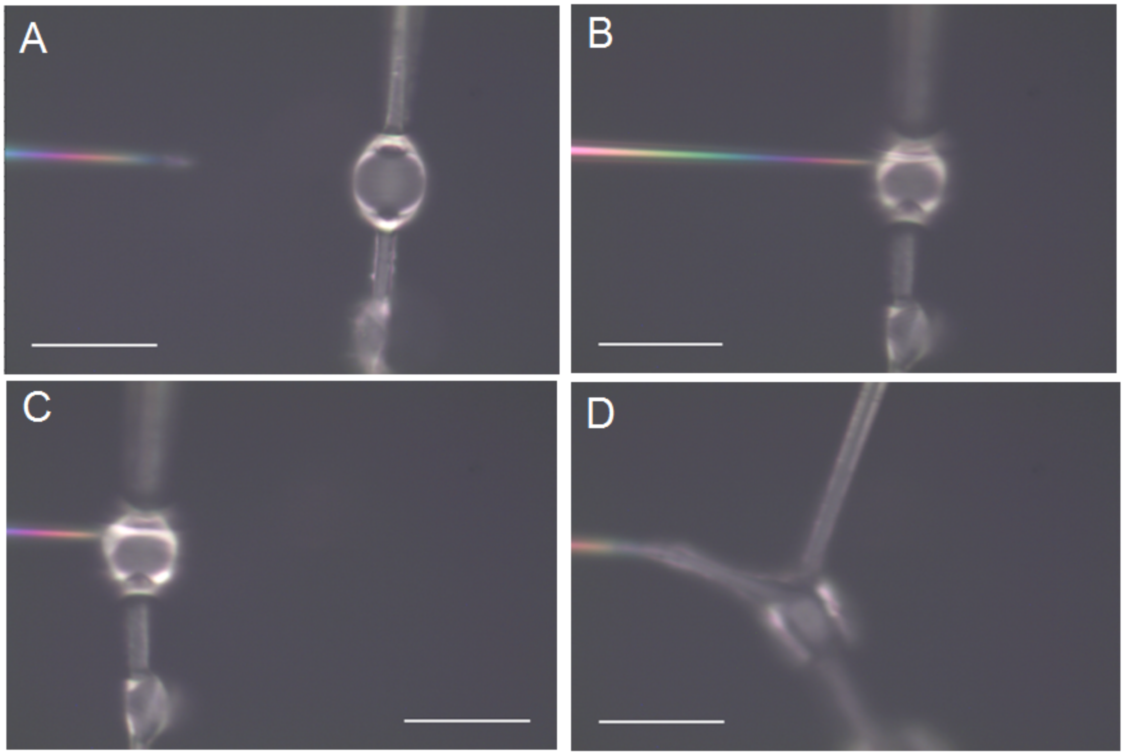

Figure S8: Sequence of images showing an orb-web glue droplet probed by a fine-tipped needle probe. A) Probe approaching the glue droplet. B) Probe's tip just in contact with the surface of the glue droplet. C) Upon pulling back the probe, the entire thread gets pulled. The extension in the thread was greater than the field of view of the microscope. Hence, the stage was manually moved to observe the tip pull-off. D) Stretching of the glue observed. Notice the fibrous thread being pulling out of the surface of the glue droplet. All scale bars are 50 μm.
